# COVID-19 mortality and deprivation: pandemic, syndemic, and endemic health inequalities

**DOI:** 10.1016/S2468-2667(22)00223-7

**Published:** 2022-11-01

**Authors:** Victoria J McGowan, Clare Bambra

**Affiliations:** Population Health Sciences Institute, Newcastle University, Newcastle upon Tyne, UK; Fuse–The Centre for Translational Research in Public Health, Newcastle Upon Tyne, UK

## Abstract

COVID-19 has exacerbated endemic health inequalities resulting in a syndemic pandemic of higher mortality and morbidity rates among the most socially disadvantaged. We did a scoping review to identify and synthesise published evidence on geographical inequalities in COVID-19 mortality rates globally. We included peer-reviewed studies, from any country, written in English that showed any area-level (eg, neighbourhood, town, city, municipality, or region) inequalities in mortality by socioeconomic deprivation (ie, measured via indices of multiple deprivation: the percentage of people living in poverty or proxy factors including the Gini coefficient, employment rates, or housing tenure). 95 papers from five WHO global regions were included in the final synthesis. A large majority of the studies (n=86) found that COVID-19 mortality rates were higher in areas of socioeconomic disadvantage than in affluent areas. The subsequent discussion reflects on how the unequal nature of the pandemic has resulted from a syndemic of COVID-19 and endemic inequalities in chronic disease burden.

## Introduction

The COVID-19 pandemic has occurred against a back-drop of existing social and economic inequalities in non-communicable diseases.^1^ Although the effects of COVID-19 have been examined across various dimensions of health inequalities such as age,^2–5^ disability,^6^ gender,^7–10^ race and ethnicity,^5,9,11–16^ sexuality,^17^ occupation,^18^ and socioeconomic status,^16,19^ geographical inequalities by area-level deprivation have been relatively less explored (yet notable early exceptions include work in the USA by Chen and Krieger^20^ or in Europe such as by Daras and colleagues^21^ or by Niedzwiedz and colleagues^22^). The links between place and health have been long established in the scientific literature,^23,24^ and endemic geographical inequalities in health have been widely documented—eg, in England,^25,26^ and in other European countries.^27,28^ There are also notable geographical inequalities in health within low-income and middle-income countries—in Chile,^29^ in India,^30,31^ and in Malawi, South Africa, Eswatini, Zambia, and Zimbabwe.^32^ These heath inequalities are entrenched and in some countries are increasing over time.^33^

Given these marked, endemic health inequalities, we sought to examine whether COVID-19 mortality rates also showed similar geographical patterns. Research in England, for example, has found large regional inequalities with high rates of COVID-19 deaths in the most deprived northern regions.^34^ However, there has been no assessment of whether there is an association between COVID-19 mortality and deprivation across different countries, in different stages of the pandemic, or at different geographical scales. Therefore, this Review explores what is known about geographical inequalities in COVID-19 mortality rates. It aims to identify and analyse the association between deprivation and COVID-19 mortality rates globally by reviewing studies from any country, at any stage of the pandemic, and at any geographical scale. The discussion draws on these findings and the wider place and health literature to reflect on how the unequal nature of the pandemic has resulted from a syndemic of COVID-19 and endemic inequalities in chronic disease burden.

## Methods

The protocol has been published elsewhere.^35^ Our inclusion and exclusion criteria was ordered by population, concept, and context.

All titles and abstracts were screened by VJM, using Rayyan QCRI,^36^ and relevant papers were retrieved and assessed for inclusion. Ambiguous studies were discussed with CB. Following guidance for conducting scoping reviews by Peters and colleagues,^37^ data pertaining to population, location, and outcomes were extracted from full-text versions of included studies using standardised extraction forms. A formal assessment of study quality and risk of bias was not done.^38–40^

A process of charting the results was done, in which a descriptive summary of the study characteristics and findings pertaining to the Review topic were tabulated (appendix pp 8–47). Studies were tabulated by WHO region and their findings narratively synthesised (on the basis of the direction of effect determined by whether studies showed significant results, dichotomised into a positive significant association *vs* no or a negative association) to summarise the global evidence base on geographical inequalities in COVID-19 mortality.

## COVID-19 mortality and deprivation

### Overview of the findings

95 primary studies were included in this Review. Initially, 22 190 citations were retrieved from the four databases searched. All records were uploaded to Rayyan and after deduplication, a total of 13 930 unique citations were subject to title and abstract screening. This process resulted in 360 full texts being screened for inclusion of which 145 were selected for full-text review, and 50 of those were excluded with reasons provided (appendix pp 48–52). The process of study inclusion and exclusion is depicted in the Preferred Reporting Items for Systematic Reviews and Meta-Analyses flow chart^41^ (appendix p 53).

Included studies were in the following five WHO regions: the Americas (n=72); Europe (n=20); Africa (n=1); South-East Asia (n=1); and the Western Pacific (n=1). No studies were identified from the Eastern Mediterranean region. The results and locations of the different studies are summarised in the appendix (pp 8–47) and a map of the global distribution of included studies is also provided (appendix p 54).

Although study outcomes (ie, COVID-19 mortality) were homogeneous, measures of socioeconomic disadvantage varied across the studies (including poverty, income, education, [un]employment, deprivation, social vulnerability, and car and computer ownership). There was also great variation in the timepoints for data used in the studies ranging from a few weeks in the early stages of the pandemic to the whole first year. Considering these variations in socioeconomic measures and time periods is important when interpreting the results of the studies.

91% (86 of 95) of the studies^20,21,42–125^ found that COVID-19 mortality was significantly higher in areas of social disadvantage than in affluent areas. Only a small minority of studies (n=9)^126–134^ showed mixed results. Three studies^126–128^ showed high mortality rates regardless of deprivation and one of these studies^127^ noted that mortality rates shifted back and forth between deprived and affluent areas over time. Four studies^129–132^ showed no association between markers of economic disadvantage and COVID-19 mortality; however, two of these studies^129,130^ found higher rates among areas with high minority ethnic populations than in areas with low ethnic minority populations, one study found a substantial relationship between COVID-19 case fatalities and poverty,^131^ and one found greater area income inequality was associated with higher infections but not deaths.^132^ Two studies^133,134^ showed higher COVID-19 mortality rates in more advantaged areas.

### Region of the Americas

72 studies were from the WHO region of the Americas, of which 55 studies^20,42–92,127,129,130^ were from the USA. 52 of the US studies showed that COVID-19 mortality rates were higher in areas of social disadvantage than in affluent areas. For example, Oronce and colleagues^79^ examined the relationship between state-level COVID-19 mortality and the Gini index in the early phase of the pandemic (ie, from Jan 22 to April 13, 2020), observing that states with higher income inequality had higher deaths than states with lower income inequality. At the county level, Al Rifat and Liu^42^ found a positive correlation between social vulnerability and COVID-19 mortality during the first year of the pandemic (ie, from Jan 20, 2020, to Jan 20, 2021). Chen and Krieger^20^ found that COVID-19 death rates were consistently higher in the most disadvantaged counties and neighbourhoods (up to May 5, 2020). Three US studies showed mixed results.^127,129,130^ For example, Neelon and colleagues^127^ examined mortality rates from March 15 to Dec 31, 2020 and found that, although more socially vulnerable counties had higher death rates early in the pandemic (ie, up to May, 2020), less socially vulnerable areas had higher rates later (ie, by October, 2020).

In Brazil, positive associations were found between social disadvantage and COVID-19 mortality rates in all 12 studies conducted at different timepoints.^93–103,126^ For example, de Souza and colleagues^97^ (up to May 6, 2020) and Silva and Ribeiro-Alves^101^ (up to May 23, 2020) found that people living in high-income areas were more at risk of COVID-19 infection, but those living in more deprived areas had higher death rates. Studies from Chile,^104^ Columbia,^105^ and Mexico^106,107^ all observed positive associations between indicators of social disadvantage and COVID-19 mortality rates. For example, Benita and Gasca-Sanchez^106^ examined COVID-19 deaths at the municipality level in Mexico from June 1 to Aug 22, 2020, and found that income inequality was strongly associated with mortality. In Peru however, Dorregaray-Farge and colleagues^131^ did not observe a significant association between poverty and mortality at the district level in Metropolitan Lima from March 18 to Sept 30, 2020, but found a significant correlation between COVID-19 case fatalities and poverty.

### European region

There were 20 studies conducted within the WHO European region. All studies from the UK (n=9),^20,108–115^ Germany (n=3),^116–118^ France (n=1),^119^ Hungary (n=1),^120^ Italy (n=1),^121^ and Switzerland (n=1)^122^ showed positive associations between area-level indicators of socioeconomic disadvantage and COVID-19 mortality rates. For example, Chaudhuri and colleagues^112^ examined inequalities in COVID-19 mortality rates at the local authority level in England from March 1 to April 16, 2020, and found that the most deprived areas had significantly higher COVID-19 mortality compared with the least deprived areas. In Germany, Hoebel and colleagues^116^ found that COVID-19 mortality rates increased faster in more deprived districts between September, 2020, and March, 2021, than in less deprived districts. Ginsburgh and colleagues^119^ observed more deaths in departments with greater income inequality in France, from March 1 to Sept 3, 2020, than in departments with less income inequality. In Hungary, Oroszi and colleagues^120^ found a strong positive relationship between mortality and deprivation at the municipality level up to April 13, 2021. Di Girolamo and colleagues^121^ found that in the Emilia-Romagna region of northern Italy, COVID-19 mortality rates were high in the most disadvantaged census blocks from March 1 to April 31, 2020. In Switzerland, Riou and colleagues^122^ found that COVID-19 mortality was high in neighbourhoods with a low socioeconomic status up to April 14, 2021.

Two studies from Spain,^133,134^ however, showed that COVID-19 mortality was associated with socioeconomic advantage and one showed increased area-level income inequality was associated with high infections but not deaths.^132^ For example, Garcia^134^ examined COVID-19 mortality rates across the 17 autonomous communities of Spain up to May 23, 2020, and found that a 1% increase in the gross domestic product per capita was associated with a 3·1% increase in COVID-19 mortality. One study from Sweden^123^ found no significant association between COVID-19 mortality and area-level income or education.

### African region

Only one study from the WHO Africa region was found. This South African study by Hussey and colleagues^124^ identified a socioeconomic gradient in COVID-19 mortality at the subdistrict level in Cape Town up to Feb 24, 2021.

### South-East Asia region

Only one study from the WHO South-East Asia region was included.^128^ This study by Middya and Roy^128^ examined geographical inequalities in COVID-19 deaths at the district level in India up to Feb 24, 2021. They found mixed results with high mortality rates in affluent areas in the COVID-19 death hotspots of eastern and western India, but a strong negative relationship between COVID-19 death rates and education in hotspots of eastern, central, and southern regions of India.

### Western Pacific region

One study from the WHO Western Pacific region was included. Yoshikawa and Kawachi^125^ observed high COVID-19 mortality rates in areas with the greatest socioeconomic disadvantage at the prefecture (ie, regional) level in Japan for the first year of the pandemic up to February, 2021.

## Pandemic, syndemic, and endemic health inequalities

Overall, the vast majority of studies showed inequalities by various measures of area-level deprivation at differing geographical scales, from neighbourhood to region. Evidence of area-level socioeconomic inequalities in COVID-19 mortality rates were found in four of the six WHO world regions (ie, the Americas, Europe, Africa, and the Western Pacific). Most of the studies were conducted in the Americas and Europe with only some from other WHO regions; no studies were found for the Eastern Mediterranean region and the single study for South-East Asia had mixed results. The scarcity of studies on the association between area-level deprivation and COVID-19 mortality outcomes from most of the countries on the planet speaks to our lack of knowledge about inequalities in most places, and the poor investment in researching this topic.

Our findings are broadly in keeping with extensive data on area-level inequalities in other pandemics. For example, area-level inequalities were documented in the 2009 H1N1 influenza pandemic in England^135^ and there are well documented inequalities by deprivation in seasonal winter influenza among both adults and children.^136,137^ Research into the Ebola virus disease outbreaks in west Africa and the Democratic Republic of the Congo has also found that transmission was high in the most impoverished communities and that most of the spread originated in low socioeconomic status areas.^138^ Similarly for the congenital Zika syndrome pandemic in Brazil, there is evidence of a strong association between prevalence rates and living conditions.^139^ Even historical research into the 1918 Spanish influenza pandemic has documented area-level inequalities in mortality related to deprivation (eg, household size and income).^140–142^ Similarly, our results reflect the findings of reviews of socioeconomic inequalities in COVID-19 at the individual level. For example, an international systematic review of inequalities in COVID-19 outcomes found that ethnic minorities and low socioeconomic groups had high risks of COVID-19 infection, hospitalisation, confirmed diagnosis, and death.^143^ The long-term increases in health inequalities, already under way in some countries (for example in mortality), might be exacerbated by the pandemic.^33^

Understanding the relationship between deprivation and COVID-19 mortality rates is multifaceted. The COVID-19 pandemic has been described as a syndemic pandemic.^1,144^ Originating in anthropology, a syndemic describes “a set of closely intertwined and mutual enhancing health problems that significantly affect the overall health status of a population within the context of a perpetuating configuration of noxious social conditions”.^145^ Deprivation—which is an area measure of poverty, low income, and a reflection of the wider social determinants of health (such as housing, working conditions, unemployment, health-care access, etc)— results in multiple, interacting, and additive adverse risk factors for COVID-19 mortality.^1^ These can be summarised by way of four inter-related pathways: unequal exposure, unequal transmission, unequal vulnerability, and unequal susceptibility.^144,146^

Unequal exposure results from variations in the ability to shield from infection. For example, people in more deprived areas are more often in jobs that are less amenable to remote working and so they benefit less from lockdown restrictions than those able to work from home.^144^ Unequal transmission is the increased risk of infection spreading within the community for people living in more deprived areas. For example, self-isolation if infected is hard in overcrowded houses and in more urban areas and areas of high population density.^147^ Unequal vulnerability is the increased risk of mortality from the high burden of non-communicable diseases in socioeconomically disadvantaged areas. For example, key clinical risk factors for adverse COVID-19 outcomes such as respiratory disease, obesity, or heart diseases are all higher in more deprived areas.^148^ Unequal susceptibility arises from the increased risk of more severe disease for people from disadvantaged backgrounds, due to weakened resilience from chronic exposure to the social determinants of health. For example, studies have found that adverse psychosocial circumstances increase immunosuppression—influencing the onset, course, and outcome of disease.^149^

Also, of potential relevance to understanding our findings is the health inequalities framework—originally proposed by Diderichsen and colleagues^150^ and elaborated on by Katikireddi and colleagues^151^ in relation to ethnic inequalities in the COVID-19 pandemic. This approach articulates an additional three pathways to inequality in the pandemic including: (1) the differential social consequences of COVID-19 (eg, disability that results in job loss and future loss of earnings due to poor health), (2) the differential effectiveness of pandemic control measures (eg, their effect on risk of exposure, vulnerability, and consequences might be different for different communities), and (3) the differential adverse consequences of control measures (eg, the economic effects of pandemic control measures [such as loss of income] might also disproportionately affect disadvantaged groups more). These could all also apply when considering the potential causes of area-level inequalities in the pandemic that our Review has summarised.

Our Review was limited to studies showing data from the first 2 years of the pandemic (2020–21) and most of the data comes from the first 18 months. As such, we have not been able to capture the extent of inequalities in COVID-19 mortality rates by deprivation after the start of vaccination programmes. This information could be particularly important in high-income countries in which vaccines have been widely distributed. However, despite vaccines often being provided in these contexts at no or low cost, there have been clear inequalities by deprivation in rates of uptake.^152^ Indeed, vaccine uptake and unequal health-care treatment more generally (eg, hospital care, respirators, and antivirals) can be considered to be an additional pathway leading to pandemic inequalities.^146^ The unequal distribution of vaccine uptake means that future COVID-19 outbreaks can be expected to be geographically concentrated in areas and regions of high deprivation (and low uptake). In countries with low vaccine coverage (including the majority of low-income and middle-income countries) we can also expect inequalities in COVID-19 mortality to continue—albeit with a lower overall death rate given the current dominance of the omicron strand. New variants could of course further exacerbate the unequal pandemic.

The inequalities by deprivation in COVID-19 mortality that we have summarised in this Review reflect wider patterns of endemic geographical inequalities in health.^24^ There are well documented, long-standing health inequalities within and between places, operating at different scales: from the life expectancy gap of 9 years between men living in the most and least deprived neighbourhoods of England, to the excess mortality in the west of Scotland, the US mortality disadvantage, or regional inequalities in life expectancy across India.^31,153^ These endemic health inequalities arise from a mixture of the nature of the places themselves and the characteristics of the populations living within them (sometimes referred to as compositional and contextual factors).^24^ Health inequalities by place arise from differences in compositional risk factors such as demographics (eg, age, sex, and ethnicity), health-related practices (eg, smoking, alcohol consumption, physical activity, diet, drug use, and gambling), and socioeconomic characteristics (eg, income, education, and occupation) of the people living within the area (ie, neighbourhood, town, city, or region).^24^ They are also influenced by the contextual nature of the place in terms of the socio-spatial determinants of health including the economic (eg, area-level poverty rates, unemployment rates, wages, types and quality of work, and job availability), social (eg, services such as childcare, food supply, health care, housing or schools, and social cohesion), and physical (eg, air pollution, access to green spaces, and the built environment) environments.^24^ These factors are also influenced by the macro political, economic, and policy conditions operating at national and, increasingly, international levels.^153^ The fact that patterns of COVID-19 follow these endemic health inequalities is unfortunately no surprise as they too reflect these underpinning pathways.

Although there are various immediate tools available to public health policy makers for reducing inequalities in COVID-19—most notably increasing vaccine availability and uptake in more deprived global regions and areas (eg, via mobile vans, door-to-door outreach, and over-coming reasons for hesitancy among more disadvantaged communities such as low social trust or cultural norms, via education, etc)^154,155^—ultimately improving our future pandemic preparedness requires long-term solutions to health inequalities through tackling the social determinants of health. Reducing inequalities in pandemics requires reducing inequalities in the underpinning endemic inequalities in chronic disease and the social determinants of health. This task is not straightforward or quickly achieved but there is evidence of effective policy actions in different global contexts—from the expansion of civil rights in the USA in the 1960s to the democratisation of Brazil in the 1980s as well as the English Heath Inequalities strategy of the 2000s and the reunification of Germany in the 1990s.^156^

## Strengths and limitations

We followed established scoping review methods and carried out a systematic, international, and wide-ranging search for studies of inequalities by area-level deprivation in COVID-19 mortality during the first 2 years of the pandemic. We included studies from any country, at any geographical scale, and used a broad and inclusive measure of deprivation. However, our study is also subject to some important limitations.

Firstly, although our Review examined studies published up to July, 2022, the majority of studies used data from 2020. As such they relate to the first-known variant, alpha, beta, and delta variants only. They do not examine inequalities in the more recent omicron variant, nor do they capture post-vaccine mortality rates.

Secondly, we used COVID-19-specific mortality, as opposed to a measure of excess mortality, and this approach could have underestimated the effects of area-level deprivation on mortality especially in countries with less established testing and reporting processes. Similarly, we did not examine other measures of adverse COVID-19 outcomes such as case rates, hospitalisations, or symptom severity. There was also potential heterogeneity in the COVID-19 mortality metrics used by different countries (eg, differences in the criteria for attributing a death to COVID-19). This discrepancy might also have been affected by different testing capacities or reporting policies between countries. Moreover, some differences in the mortality monitoring systems between countries could affect the availability of studies in the period covered by this Review.

Thirdly, this paper is a scoping review, not a full systematic review, and as such no critical appraisal was conducted, and screening and data extraction was done by only one reviewer. We have found a large evidence base (at least for some regions) and therefore recommend a full systematic review with meta-analysis to be conducted soon. Our Review also had other methodological limitations such as only including papers published in English, papers published as full papers, and papers of full populations (thereby excluding those studies based on random samples, which could have inadvertently excluded studies from some countries more than others). We also made some small refinements to the inclusion criteria originally set out in our protocol: (1) studies were required to include data from whole populations rather than just subsections (eg, women, children, and ethnic minorities); (2) case fatality rates were excluded due to concerns over inequalities in testing;^46,109,111,157^ and (3) studies had to show a measure of socioeconomic deprivation (eg, indices of multiple deprivation, percentage living in poverty, proxy factors [including the Gini coefficient as deprivation tends to be highly correlated with income inequality], employment rates, or housing tenure).^158^ Notably, another limitation is that in our summary of effect direction, we relied on whether the authors reported significant findings. Any full systematic review could also use other approaches.^159^

Fourthly, although we have identified relationships at the area level we cannot assume that our findings hold true at the individual level: the ecological fallacy.^160^ However, we make no claims to causality within this Review.

Fifthly, the included studies did not show the intersectional relationship between area-level deprivation and gender (or other axes of inequalities such as ethnicity). So, we were unable to report on additional subgroup differences (eg, whether mortality rates differed between men and women in more or less deprived areas). This lack of intersectionality in geographical studies has been noted previously and is further highlighted in our Review.^161^

Finally, there are also limits to the evidence base itself: the vast majority of studies were from the USA and so generalisability to other countries might be limited. There was a clear dearth of studies conducted in other regions especially those outside high-income regions. Further, many of the included studies were conducted at a large geographical scale (eg, region, county, or municipality) when analysis of smaller-level geographies (ie, neighbourhoods) might allow a more precise estimation of the extent of area-level inequalities in COVID-19 mortality. However, large units can help in considering the way different institutions and services might be contributing to inequalities whereas small areas, because they are potentially a better proxy for individual-level exposures, might provide insight into more proximal pathways.

## Conclusion

This Review has found extensive evidence of inequalities in COVID-19 mortality rates by area-level deprivation across the world. The pandemic has been an unequal experience with high mortality rates in the most deprived places and communities. These inequalities can be understood as a syndemic, arising from endemic inequalities in the social determinants of health. Reducing these inequalities—and those that might arise from future pandemics—requires long-term action to reduce inequalities in health and wealth. Future research and data collection should focus on improving surveillance systems by, for example, integrating measures of inequalities into the WHO Mortality Database.

## Search strategy and selection criteria

Searches were conducted in four databases (host sites): MEDLINE (Ovid), Embase (Ovid), Science Citation Index Expanded, and Social Science Citation Index (Web of Science) from July 11 to July 15, 2022. The inclusion criteria were guided by the population, concept, and context mnemonic. The population was the total population of the study location; the concept was geographical inequalities in COVID-19 mortality within countries; and the context was any country at any geographical level (eg, neighbourhood, town, city, municipality, or region). Only peer-reviewed studies, from any country, written in English, and published during the first two and a half years of the COVID-19 pandemic (ie, from Jan 1, 2020, to July 11, 2022) were included. Our full search strategy is listed in the appendix (pp 3–7).

Search terms included (SARS-Cov-2 or 2019-nCOv or COVID-19 or coronavirus or exp COVID-19/) and (fatalit* or death* or mortalit* or exp death/ or exp mortality/) and (socioeconomic or SES or education* or employment or income or occupation* or poverty or class or depriv* or disadvantage* or social class or social factors or economic or unemployment or ethnic* or rac* or minorit* or exp socioeconomic factors/) and (area* or geo* or place* or neighbourhood* or region* or count* or ward* or cit* or district* or municipal* or province* or state* or communit* or count* or town* or district* or census or post* or zip or spatial or metropolitan or depriv* or environ*).

### Inclusion criteria

- The publication is a peer-reviewed empirical study published in English
- Population; the publication focuses on COVID-19 deaths or mortality rates from the total population of the study location
- Concept, the publication disaggregates COVID-19 mortality rates by measures of the area’s socioeconomic deprivation (eg, Gini index, poverty, income, education, unemployment and employment, deprivation, social vulnerability, and car and computer ownership)
- Context, the publication covers concepts at any geographical level (eg, neighbourhood, town, city, municipality, and region)

### Exclusion criteria

- The publication is a conference proceeding, editorial, letter, comment, erratum, survey, note, or doctoral thesis; or does not meet one or more of the key elements of population, concept, and context
- The publication focuses on COVID-19 hospitalisations, incidences, cases, case fatalities, symptoms and severity, or infection rates
- The publication does not include COVID-19 mortality disaggregated by an area measure of socioeconomic deprivation
